# Early and Mid-Term Outcomes of Primary Repair After Atrioventricular Canal Defect: A Single-Center Eight-Year Experience

**DOI:** 10.7759/cureus.45304

**Published:** 2023-09-15

**Authors:** Rupesh Kumar, Vikram Halder, Soumitra Ghosh, Shyam Thingnam, Harkant Singh, Anand K Mishra, Sachin Mahajan, Pankaj Aggarwal, Aduri Raja S Dutta, Amit Mishra

**Affiliations:** 1 Department of Cardiothoracic Surgery, Postgraduate Institute of Medical Education and Research, Chandigarh, IND; 2 Department of Cardiothoracic Surgery, U. N. Mehta Institute of Cardiology and Research Centre, Ahmedabad, IND; 3 Department of Cardiology, Postgraduate Institute of Medical Education and Research, Chandigarh, IND

**Keywords:** mortality, surgical outcomes, surgical repair, down syndrome, atrioventricular septal defect

## Abstract

Background/aim: Surgical repair techniques and management of patients with atrioventricular septal defect (AVSD) have progressed over the last few decades. Early and definitive interventions have become the choice of treatment for these patients. Based on this background, we aimed to review the early and mid-term outcomes of primary AVSD repair.

Methods: A total of 53 patients with a mean age of 3.45 ± 5.67 years underwent definitive repair for AVSD between January 2014 and June 2021. The clinical data including age, type of defect, associated co-anomalies, symptoms, pulmonary hypertension, etc. were collected and assessed retrospectively. Mitral regurgitation (MR) as a clinical outcome was assessed at 0, 1, 2, and 5 years.

Results: Among the recruited patients, 35 (66.1%) were male and 18 (33.9%) were female. Of 53 patients, repair for the complete defect was done in 38 (71.69%) patients, repair for intermediate/partial defect was done in 15 (23.1%) patients, and one patient underwent repair for incomplete type. Other associated co-anomalies were anterior mitral leaflet (12 (22.6%)), atrial and ventricular septal defect (VSD) (30 (56.6%)), and patent ductus arteriosus (PDA) (11 (20.8%)). Different procedures for surgical repair included patch closure, cleft repair, and polytetrafluoroethylene (PTFE) VSD closure. After repair, the mean follow-up period was 46.73 ± 27.37 months. Overall mortality was 3.78% (2/53), and two patients underwent reintervention due to symptomatic severe MR.

Conclusions: A definitive and timely correction of AVSD shows satisfactory early and mid-term results.

## Introduction

Atrioventricular septal defects (AVSD) cover an array of congenital heart malformations characterized by the common atrioventricular junction and deficient septation [1]. It is also known as an atrioventricular canal defect or endocardial cushion defect, accounting for 5% of all congenital malformations. It occurs due to the abnormal development of endocardial cushions. Based on the valve leaflets arrangement, AVSD is categorized into three types: complete (ostium primum atrial septal defect (ASD) and nonrestricted ventricular septal defect (VSD)), incomplete/partial (ostium primum ASD, cleft in the mitral leaflet), and transitional/intermediate defect (ostium primum ASD and restricted VSD). Complete AVSD is further classified into Rastelli A, B, and C as per the bridging of the left superior leaflet [2]. Clinically, patients with AVSD are represented with symptoms of heart failure and pulmonary hypertension as fast breathing, sweating during feeding, and shortness of breath. It has a strong association with trisomy 21 or Down syndrome, with 15-20% of all affected patients [3]. Two-dimensional (2D) echocardiography is a diagnostic modality of choice. The surgical management of AVSD is the mainstay for treatment. Historically, Dr. Lillehei carried out the first AVSD repair in 1955 [4]. The different methods of primary repair of AVSD include the single patch technique, double patch technique, and modified single patch technique. It is necessary to correct the defect within three to six months to prevent pulmonary hypertension. Other associated techniques are patch closure, cleft repair, and polytetrafluoroethylene (PTFE) VSD closure [5]. Surgery is done through midline sternotomy and cardiopulmonary bypass. Prolonged intensive care unit stay, complete heart block, chylothorax, and postoperative pulmonary hypertensive crisis are common complications after repair. Left AV valve regurgitation and left ventricular outflow tract obstruction are the common long-term complications for which reoperation or reinterventions are needed [6-8]. Based on this background, the present study aimed to review early and midterm outcomes of primary repair after AVSD in 53 patients over a period of eight years at a single center.

## Materials and methods

Patients

A total of 53 patients with AVSD were diagnosed or referred to undergo primary intervention, i.e., intracardiac repair (ICR) from January 2014 to June 2021 in the Department of Cardiovascular and Thoracic Surgery of the Postgraduate Institute of Medical Education and Research, Chandigarh, India.

The patients undergoing ICR for AVSD were included in the study. Patients who were not suitable for biventricular repair or not fit for surgery due to severe pulmonary hypertension were excluded.

Ethical clearance

The study protocol was approved by an Institutional Ethics Committee of the Postgraduate Institute of Medical Education and Research, Chandigarh, India (INT/IEC/2013). Written informed consent was obtained from the representatives of the children before their enrollment in the study.

Data collection

The present study is a retrospective observational study. The data of the recruited patients were reviewed and collected from medical records such as clinical charts, 2D echocardiography reports, operation theater logbooks, surgical notes, and ICU notes. The following parameters were investigated: preoperative (age, gender, weight, height, body surface area, coronary heart disease (CHD) type, AVSD type, symptoms, associated co-anomalies, and elective/emergency surgery required), diagnosis (stenosis and regurgitation), and procedures, operative (X-clamp time and cardiopulmonary pass time). All the patients were subjected to follow-up and their post-operative outcomes, i.e., mitral regurgitation (MR) at 0, 1, 2, and 5 years, New York Heart Association (NYHA) functional status, and mortality were recorded.

Statistical analysis

Data were expressed as mean with standard deviation or number and percentage of the patients. The analysis was performed using SPSS Statistics version 22 (IBM Corp. Released 2013. IBM SPSS Statistics for Windows, Version 22.0. Armonk, NY: IBM Corp.).

## Results

Baseline characteristics

The mean age of the recruited children was 3.45 ± 5.67 years, out of which age of 38 (71.7%) patients were <5 years, seven (13.2%) patients were in the age range of 5-10 years, and eight (15.1%) patients aged >10 years. Among the recruited patients, 35 (66.1%) were male and 18 (33.9%) were female. The mean weight, height, and body surface area of the recruited children were 12.19 ± 15.74 kgs, 85.7 ± 36.92 cms, and 0.58 ± 0.44 m2, respectively. Furthermore, clinical symptoms such as fast breathing were present in 40 (75.4%), growth retardation in 44 (83.01%), and shortness of breath in 49 (92.45%) children. The baseline characteristics are summarized in Table 1.

**Table 1:** Baseline characteristics of the enrolled subjects SD: standard deviation, n: number of patients, %: percentage

Parameters (Mean ± SD)	Patients (n=53)
Age, years	3.45 ± 5.67
- <5, n (%)	38 (71.7)
- 5-10, n (%)	07 (13.2)
- >10, n (%)	08 (15.1)
Sex, n (%)	
Male	35 (66.1)
Female	18 (33.9)
Weight, kg	12.19 ± 15.74
Height, cm	85.7 ± 36.92
Body surface area, m^2^	0.58 ± 0.44
Symptoms, n (%)	
Shortness of breath	49 (92.4)
Growth retardation	44 (83.0)
Fast breathing	40 (75.4)
Respiratory failure (preop intubated)	4(7.5)

Diagnostic features

Out of 53 patients, 48 (90.5%) patients underwent AVSD repair for complete defect, four (7.5%) for intermediate, and one patient for partial type. Among the patients with complete AVSD, 37 (77.0%) represented Rastelli type A. Furthermore, echocardiography diagnosis revealed associated co-anomalies such as anterior mitral leaflet cleft in 12 (22.6%) patients, ASD and VSD in 30 (56.6%) patients, and patent ductus arteriosus (PDA) in 11 (20.8%) patients. Notably, eight (15.09%) of children had Down syndrome. Furthermore, seven (14.6%) patients were diagnosed with tricuspid regurgitation, nine (17%) with aortic valve (AV) regurgitation, 12 (22.6%) with pulmonary stenosis, and 16 (30.2%) with pulmonary hypertension. The diagnostic features are recorded in Table 2.

**Table 2:** Diagnostic features AVSD: atrioventricular septal defect, CHD: congenital heart disease, AML: anterior mitral leaflet cleft, ASD: atrial septal defect, VSD: ventricular septal defect, PDA: patent ductus arteriosus, TR: tricuspid regurgitation, DORV: double outlet right ventricle, PS: pulmonary stenosis, AV: aortic valve regurgitation, n: number of patients, %: percentage

Parameters, n (%)	Patients (n=53)
Type of AVSD	
Complete	48 (90.56)
Intermediate	4 (7.5)
Partial	1 (1.8)
Complete AVSD	
Rastelli type A	37 (77.0)
Rastelli type B	7 (14.5)
Rastelli type C	4 (8.3)
Down Syndrome	08 (15.1)
Additional co-anomalies	
AML	12 (22.6)
VSD	30 (56.6)
ASD	30 (56.6)
DORV	06 (11.3)
PDA	11 (20.8)
TR	
Mild	2 (3.8)
Moderate	2 (3.8)
Severe	3 (5.7)
PS	
Mild	5 (41.6)
Moderate	3 (25.0)
Severe	4 (33.3)
Pulmonary hypertension	16 (30.2)
Mild	4 (25.0)
Moderate	2 (12.5)
Severe	8 (50.0)
AV regurgitation	9 (17)

Surgical procedures

Forty-nine (92.5%) patients underwent elective surgery, whereas four (7.5%) underwent emergency surgery. Depending on the diagnostic features and condition of the patients, they underwent a series of procedures including pericardial patch closure of ostium secundum (OS) ASD in five (9.4%), cleft closure in nine (17%), PTFE patch closure of OS ASD in two (3.7%), PDA ligation in three (5.7%), and patent foramen ovale (PFO) closure in two (3.8%) patients (Table 3).

**Table 3:** Surgical procedures PTFE: polytetrafluoroethylene, ASD: atrial septal defect, PDA: patent ductus arteriosus, PFO: patent foramen ovale, SD: standard deviation, n: number of patients, %: percentage, SD: standard deviation

Parameters, n (%)	Patients (n=53)
Surgery	
Elective	49 (92.5)
Emergency	04 (7.5)
Type of surgery	
Emergency	04 (7.5)
Single patch repair	8 (15.0)
Modified single patch repair	5 (9.4)
Double patch repair	40 (75.4)
Pericardial patch closure of ostium secundum ASD	5 (9.4)
Additional cleft repair	09 (17)
PTFE patch closure of ostium secundum ASD	2 (3.7)
PDA ligation	03 (5.7)
PFO closure	02 (3.8)
Intraoperative parameters (mean ± SD)	
Aortic cross clamp, min	119.61 ± 52.23
Cardiopulmonary bypass, min	144.43 ± 71.29

Intraoperative parameters

The mean times of aortic cross-clamp and cardiopulmonary bypass were found to be 119.61 ± 52.23 and 144.43 ± 71.29 minutes, respectively.

Postoperative outcomes

The patients were followed up for mean months of 46.73 with a standard deviation of 27.37. Overall mortality was 3.8% (2/53 patients). Of 51 patients, 17 (33.3%) patients had NYHA I, 16 (31.3%) had NYHA II, and 18 (35.2%) had NYHA III. Furthermore, the MR was assessed after surgery at 0, 1, 2, and 5 years. Seventeen (33.3%) patients were free from MR at 0 years, whereas 25 (49%) patients with mild, five (9.8%) with moderate, and four (7.8%) with severe status. The number of patients with different MR statuses is detailed in Table 4. Lastly, only two (3.8%) patients required intervention for mitral valve repair or replacement.

**Table 4:** Postoperative outcomes SD: standard deviation, n: number of patients, %: percentage, NYHA: New York Heart Association, MR: mitral regurgitation

Parameters, n (%)	Patients (n=51)
Follow-up months (Mean ± SD)	46.73 ± 27.37
NYHA	
I	17 (33.3)
II	16 (31.3)
III	18 (35.2)
MR at 0 year	
Mild	25 (49)
Moderate	05 (9.8)
Severe	04 (7.8)
No MR	17 (33.3)
MR at one year (total patients, n=47)	
Mild	23 (48.9)
Moderate	08 (17.0)
Severe	00 (0.0)
No MR	16 (34.0)
MR at two years (total patients, n=41)	
Mild	21 (51.2)
Moderate	06 (14.6)
Severe	01 (2.4)
No MR	13 (31.7)
MR at five years (total patients, n=33)	
Mild	08 (24.2)
Moderate	10 (30.3)
Severe	00 (0.0)
No MR	15 (45.4)
Mortality	02 (3.8)
Re-intervention for mitral valve repair/replacement	02 (3.8)

## Discussion

The primary aim of this retrospective study was to assess the early and mid-term outcomes after definitive repair of AVSD. AVSD is a type of congenital heart disease that is characterized by a defect in the atrioventricular junction and septation [1]. The incidence of AVSD is only 3-5% of all congenital malformations, and only 53 patients were recruited during the eight years of the study [9]. This disease is commonly diagnosed in infants and children due to the abnormal development of the endocardial cushion in the embryological phase. Substantial evidence in the literature suggests that AVSD repair is usually performed in infants [2,10]. In our study, 71.7% (38/53) of patients were aged <5 years. AVSD affects both genders equally. In 2018, Santaro et al. carried out a population-based study to estimate the sex difference for major congenital malformations in Down syndrome [11]. The authors reported the female-to-male sex ratio as 1.3:1. In contrast to these observations, the female-to-male sex ratio is approximately 1:2 in our study. Furthermore, there is a strong association of AVSD with trisomy 21 (Down syndrome). Santaro et al. reported that 17.4% of the infants with AVSD were affected with Down syndrome [11]. Similar to these findings, 15.1% of our recruited patients had Down syndrome.

AVSD varies from complete to intermediate to incomplete type of defect. In these classified types, most of the cases are of complete AVSD. An ample number of clinical studies have collected data from patients exclusively with complete AVSD. In a Lebanese study, Chehab et al. analyzed the clinical characteristics of patients with complete AVSD. Among 5.5% of diagnosed AVSD patients, the authors found that 81.7% of patients belonged to the complete AVSD type [12]. In concordance with this study, 90.5% of patients with complete AVSD were recruited in our study.

Other anomalies such as AML, ASD, VSD, PDA, AV and tricuspid regurgitation, pulmonary stenosis, etc. are associated with AVSD. During the surgical management of AVSD, the correction of these defects is also taken into consideration. AVSD is corrected surgically through the one-patch, modified one-patch, and two-patch closure techniques. Based on the clinical representation of the patient, cleft repair, OS ASD and PFO closure, and PDA ligation are carried out for the management of the disease [13]. For the same, the mean aortic cross-clamp and cardiopulmonary bypass times were 119.61 ±52.23 and 144.43 ± 71.29 minutes, respectively.

Clinically, AVSD type depends on the degree of MR and associated cardiac defects [14]. Therefore, MR was considered as an important parameter to assess the early and mid-term clinical outcomes of the study. In this study, MR was assessed at 0, 1, 2, and 5 years during the follow-up period after AVSD repair. The mean follow-up period of the patients was 46.73 ± 27.37 months. We found that 10 patients were free from MR at 0 years and 11 patients varied from mild to severe MR. In the 1st year of follow-up, there was no case of severe MR, and 31 patients suffering from mild to moderate. The number of patients with mild to moderate MR further decreased from 31 to 27 with 1 case of severe MR. During the 5th year of follow-up, only 18 patients showed mild to moderate MR with no severe MR cases. The data suggest a good early and mid-term outcome with respect to MR status in patients who underwent repair for AVSD. Our study is one of its kind that has considered MR as a clinical parameter for a follow-up study to assess the mid-term outcomes of the patients after AVSD repair.

Since the first surgical correction of AVSD in 1955, our understanding of the morphology, associated anomalies, and surgical techniques have been improved by progressive advancements in this field for the management of the disease. With these advancements, mortality after AVSD repair and incidences of re-intervention have tremendously decreased. However, mortality after repair varies in the range from 3.0% to 21.7% [15]. In accordance with this data, the mortality rate is 3.77% (2/53 patients) in our study. Evidence in the literature reported that the reoperation rates vary between 6.4% and 16.6% [15]. However, the cases of re-interventions were quite lower in our study, i.e., only two (3.8%) patients underwent mitral valve intervention, out of which one patient underwent mitral valve repair and another patient underwent mitral valve replacement. Based on all these observations, we suggest that the early and mid-term outcomes are satisfactory after AVSD repair with a lower re-intervention rate.

Limitations

It was a single-centric retrospective study with a limited number of patients. Due to the lack of evidence in the literature, very few studies were found to compare our data. Follow-up data was missing for a few patients.

## Conclusions

MR is the primary cause of re-operation in patients with AVSD. Our study showed that the patients had the freedom from any kind of re-intervention or re-operation after AVSD repair. However, the mortality rate was a little high. Therefore, we conclude that the primary repair of AVSD with patch closure technique and repair of other associated anomalies including routine cleft repair at the leaflet level, OS ASD closure, and PDA ligation results in satisfactory clinical and functional outcomes with a minimal re-intervention rate in the survivors.
